# Correction: Zheng et al. Calcium Sulfate Whiskers Dual-Enhance Mechanical and Anti-Corrosion Properties of Magnesium Phosphate Coatings. *Materials* 2026, *19*, 1032

**DOI:** 10.3390/ma19194071

**Published:** 2026-09-23

**Authors:** Yaxin Zheng, Haoran Chen, Yi Liu, Xiang Gao

**Affiliations:** 1 Institute for Composites Science Innovation (InCSI), School of Materials Science and Engineering, Zhejiang University, Hangzhou 310058, China; 22326125@zju.edu.cn (Y.Z.); 22460610@zju.edu.cn (H.C.); 2 Zhejiang Key Laboratory of Advanced Composites & Structures, Zhejiang University, Hangzhou 310058, China; 3 Ningbo Global Innovation Center, Zhejiang University, Ningbo 315100, China

**Error in Figure**

In the original publication [1], there was a mistake in Figure 7 as published. The corrected Figure 7 appears below:

**Text Correction**

There were some errors in the original publication [1]. Some texts need to be updated in Sections 3.4 and 4.

In Section 3.4, paragraph 4, the sentences “Figure 7c displays the Nyquist plots for the coated samples. All samples exhibit similar characteristics, consisting of a capacitive arc in the high-frequency region and a linear slope in the low-frequency region. This behavior is indicative of a diffusion-controlled corrosion process, where the gradient of the low-frequency slope corresponds to the coating’s resistance against ion diffusion [10]. The capacitive arc observed at high frequencies represents the charge-transfer processes occurring at the interface, which are directly related to the kinetics of electrochemical surface reactions [60]. Among the samples, C3 displays the largest capacitive arc radius in the high-frequency region. Since a larger radius signifies higher charge-transfer resistance, this result demonstrates that the incorporation of 15 wt.% CSW effectively impedes the charge-transfer steps on the steel surface, thereby significantly reducing the corrosion rate.” are updated to “Figure 7c displays the Nyquist plots for the coated samples from a representative EIS replicate. All samples exhibit similar characteristics, consisting of a capacitive arc in the high-frequency region and a linear slope in the low-frequency region. The capacitive arc observed at high frequencies corresponds to the coating layer response, governed by the coating capacitance in parallel with the coating resistance (R_f_), which reflects the integrity of the physical barrier against electrolyte permeation [60]. Among the samples, C3 displays the largest capacitive arc radius in the high-frequency region. Since a larger radius signifies higher coating resistance (R_f_), this result demonstrates that the incorporation of 15 wt.% CSW effectively enhances the physical barrier properties of the coating. It should be noted that the triplicate EIS measurements exhibited some degree of variability in the low-frequency impedance response, which is attributable to the inherent microstructural heterogeneity of spray-applied cementitious coatings (e.g., local variations in interfacial adhesion and pore distribution). Nevertheless, the fitted equivalent circuit parameters (Table 4), which integrate information across the full frequency range, consistently identify C3 as possessing the optimal balance of coating resistance (R_f_ = 157.03 Ω·cm^2^) and overall corrosion protection.”.

In Section 3.4, paragraph 5, the sentences “Figure 7d shows the Bode plots for each group of coated samples. The impedance modulus (|Z|) of all samples with different ratios exhibits typical characteristics as a function of frequency. In the low-frequency region (<1 Hz), |Z| decreases with increasing frequency, while in the high-frequency region (>10^3^ Hz), |Z| tends to stabilize. In the Bode diagram, the impedance modulus at 0.01 Hz (|Z|^0.01Hz^) is an important parameter for determining the coating’s shielding performance, with higher values corresponding to better protection against corrosive media [61]. The tested samples’ |Z|^0.01Hz^ values are ordered as follows: B is lower than C1, C2, C4, and C3. Specifically, the |Z|^0.01Hz^ value of group C3 (15 wt.% CSW) is 124.8% higher than that of the unmodified MKP control B. Furthermore, when the CSW content was ≤15 wt.%, the MKP coating’s shielding performance improved with higher content. However, when the CSW content reaches 20 wt.% (group C4), the coating’s |Z|^0.01Hz^ value significantly decreased. This decline is attributed to an excessive amount of whiskers, which disrupts the continuity of the cementitious network in the coating and thereby reduces its ability to block corrosive species [57].” are updated to “Figure 7d shows the Bode modulus plots for the same replicate presented in Figure 7c. The impedance modulus (|Z|) of all samples exhibits typical characteristics as a function of frequency. In the low-frequency region (<1 Hz), |Z| decreases with increasing frequency, while in the high-frequency region (>10^3^ Hz), |Z| tends to stabilize. In the Bode diagram, the impedance modulus at 0.01 Hz (|Z|^0.01Hz^) is an important parameter for determining the coating’s shielding performance, with higher values corresponding to better protection against corrosive media [61]. In the representative replicate shown, the |Z|^0.01Hz^ values are ordered with C3 exhibiting the highest impedance among the modified coatings. Across the triplicate measurements, two of three replicates consistently showed C3 with the highest |Z|^0.01Hz^, while one replicate showed marginally elevated low-frequency impedance for the unmodified control (B), reflecting the inherent microstructural variability of spray-applied coatings. Overall, the low-frequency impedance trend supports the enhanced shielding performance of the 15 wt.% CSW formulation.”.

In Section 4, the sentence “While the EIS measurements exhibited inter-replicate variability consistent with the microstructural heterogeneity inherent to spray-applied cementitious coatings, the overall electrochemical and mechanical evidence consistently supports 15 wt.% CSW as the optimal formulation.” has been added before the word “Ultimately”.

The authors state that the scientific conclusions are unaffected. This correction was approved by the Academic Editor. The original publication has also been updated.

## Figures and Tables

**Figure 7:**
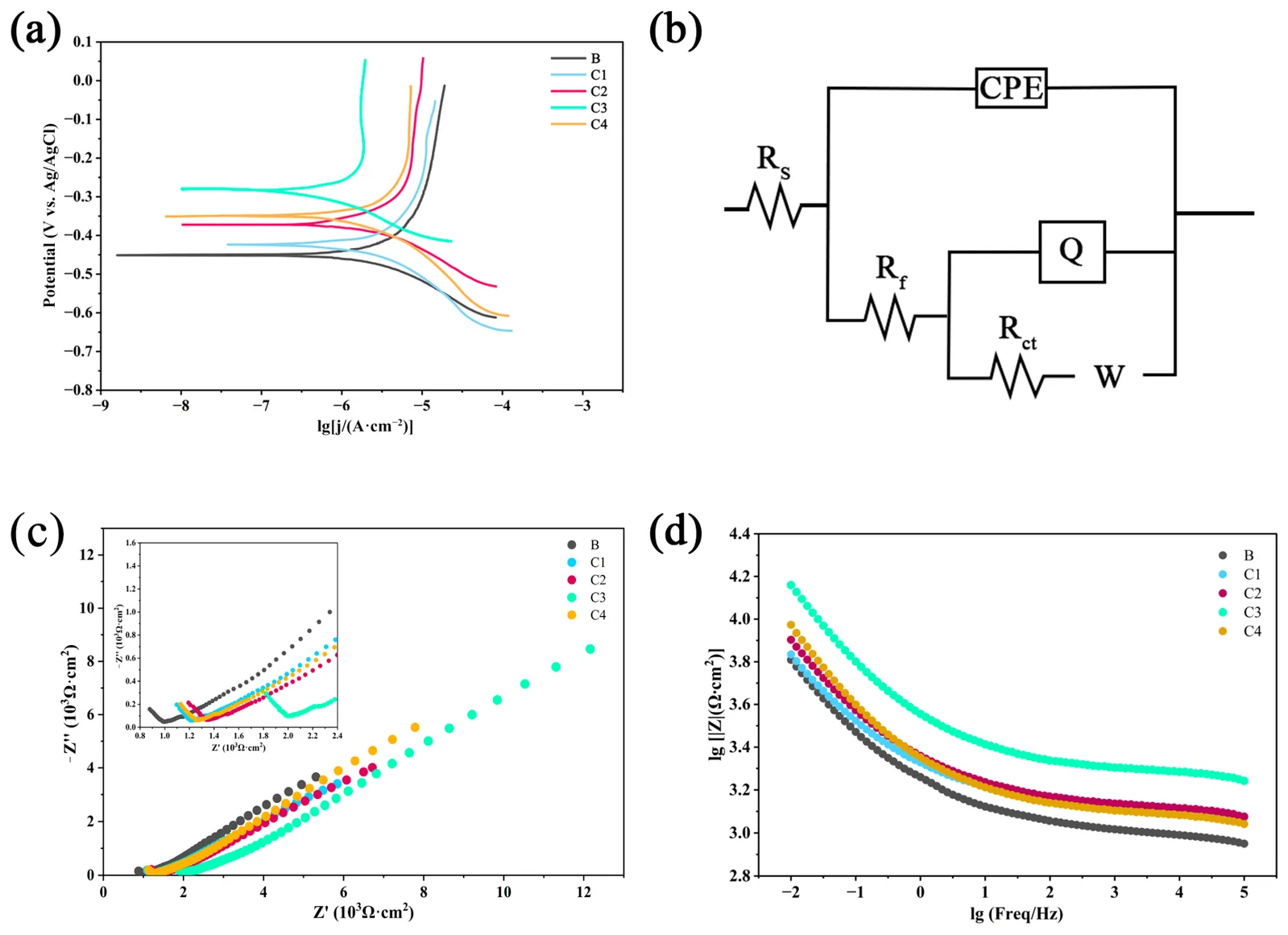
Electrochemical properties of the coatings. (**a**) Tafel curves of coating samples after 28 days of curing; (**b**) The equivalent circuit model; (**c**) Nyquist plots of coating samples and partial enlargement of the high-frequency area; (**d**) Bode plots of test samples.
